# Theta-frequency subthalamic nucleus deep brain stimulation selectively improves visual attention in Parkinson’s disease

**DOI:** 10.3389/fnagi.2026.1961905

**Published:** 2026-09-18

**Authors:** Yue Lu, Quan Zhang, Hutao Xie, Houyou Fan, Yichen Xu, Guofan Qin, Yutong Zhuang, Jiansong Huang, Anchao Yang, Yin Jiang, Jianguo Zhang

**Affiliations:** 1 Department of Neurosurgery, Beijing Tiantan Hospital, Capital Medical University, Beijing, China; 2 Beijing Key Laboratory of Neurostimulation, Beijing, China

**Keywords:** deep brain stimulation, Parkinson’s disease, subthalamic nucleus, theta-frequency, visual attention

## Abstract

**Objective:**

Visual attention deficits are common but under-recognized in Parkinson’s disease (PD), and we investigate whether theta-frequency (5 Hz) STN-DBS differentially modulates visual attention compared with high-frequency (130 Hz) stimulation in PD patients.

**Methods:**

Thirty-nine PD patients with bilateral STN-DBS perform an attentional capture task under three stimulation conditions (OFF, low-frequency, high-frequency). Patients are stratified into impaired (*n* = 21) and normal (*n* = 18) visual attention groups based on their accuracy in the AC-distractor task during OFF-stimulation, with <90% accuracy defining impairment. Behavioral accuracy and reaction time are analyzed using Bayesian mixed-effects models. P300 amplitudes and latencies are measured using electroencephalography.

**Results:**

In the impaired group, 5 Hz stimulation significantly improves accuracy in the distractor condition compared with OFF (BF > 1,000), whereas 130 Hz stimulation reduces accuracy (BF = 122.8). This behavioral pattern is paralleled by enhanced P300 amplitude and prolonged latency under 5 Hz stimulation, and suppressed P300 amplitude under 130 Hz stimulation. No significant stimulation effects are observed in the normal group.

**Conclusion:**

Theta-frequency STN-DBS selectively improves visual attention in PD with pre-existing attentional deficits, likely by enhancing top-down attentional resource allocation, while high-frequency stimulation impairs performance.

## Introduction

Parkinson’s disease (PD) is a progressive neurodegenerative disorder characterized not only by its classic motor symptoms—tremor, rigidity, and bradykinesia—but also by a broad spectrum of non-motor impairments that profoundly affect patients’ quality of life (Johansson et al., 2023; Lawson et al., 2016). Among these, cognitive deficits have received increasing attention, with nearly half of all patients developing dementia within ten years of diagnosis (Gibson et al., 2024). In particular, visual attention impairments represent a significant yet often under-recognized challenge in PD, manifesting as difficulties in visual search, reading, driving, and navigation—tasks that require the allocation of attentional resources to relevant visual information (Fiebelkorn and Kastner, 2020). These deficits not only contribute to functional disability but are also linked to more complex and debilitating symptoms such as visual hallucinations, further underscoring their clinical importance.

The neural basis of visual attention is traditionally understood as a competition between two interacting systems: a top-down, goal-directed mechanism (endogenous attention), enabling voluntary allocation of processing resources to task-relevant stimuli; and a bottom-up, stimulus-driven mechanism (exogenous attention), which automatically orients attention towards salient or unexpected sensory events (Tommasi et al., 2015). Converging evidence suggests that these mechanisms are supported by distinct but interconnected cortical networks, involving prefrontal, frontal, and parietal regions (Saffari et al., 2023). Critically, the basal ganglia, particularly the subthalamic nucleus (STN), are thought to play a cardinal role in this process by acting as a central hub for inhibiting competing responses and regulating the threshold for action (Mallet et al., 2007). This function is often conceptualized through the “hold your horses” model, where the STN, via the hyperdirect pathway, can rapidly suppress premature motor and cognitive responses to allow for more deliberate processing (Frank, 2006).

However, the precise contribution of the basal ganglia to top-down versus bottom-up attention remains an area of active investigation. Studies in PD patients, who exhibit dysfunction in the cortico-basal ganglia loops due to dopaminergic depletion, offer a unique window into this question. Using an attentional capture (AC) task, Tommasi et al. (2015) demonstrated that PD patients showed enhanced distractibility by salient, task-irrelevant stimuli—a hallmark of increased bottom-up capture. Critically, however, they attributed this primarily to a weakening of top-down control mechanisms, which normally served to antagonize bottom-up influences, rather than a primary enhancement of bottom-up processes.

Deep brain stimulation of the subthalamic nucleus (STN-DBS) is an established and highly effective treatment for the motor symptoms of advanced PD. Its therapeutic effect, particularly at high-frequency (≥130 Hz), is thought to be mediated by the suppression of pathological beta oscillations (13–30 Hz) within the basal ganglia-thalamocortical network (Blumenfeld and Brontë-Stewart, 2015; de Neeling et al., 2025; Mathiopoulou et al., 2024). Despite its motor benefits, the impact of STN-DBS on cognition is complex and frequency-dependent. While high-frequency stimulation (HFS) can impair specific cognitive functions such as response inhibition and verbal fluency, there is a growing body of evidence suggesting that low-frequency stimulation (LFS) in the theta (4–8 Hz) or beta (around 20 Hz) bands may actually enhance certain aspects of cognitive performance (Salehi et al., 2024; Ong et al., 2024). For instance, Kricheldorff et al. (2025) found that 20 Hz beta stimulation specifically improved automatic response inhibition in PD patients, as measured by a Go-NoGo task, without affecting conflict monitoring or controlled inhibition. Similarly, other studies have reported that 5 Hz or 6 Hz stimulation of the STN can improve conflict processing and working memory, respectively (Salehi et al., 2024; Xie et al., 2025). Moreover, Soh et al. (2024) provided causal evidence that the STN is transiently involved in suppressing active attentional processes following distracting events, and that this suppression could be modulated by DBS. These findings highlight the potential of low frequency stimulation as a tool to selectively modulate cognitive circuits within the STN.

Despite these advances, the differential effects of high- frequency versus low-frequency STN-DBS on the specific subcomponents of visual attention—the competition between top-down and bottom-up control—remain largely unexplored. Most prior work has focused on motor inhibition or executive functions, leaving a critical gap in our understanding of how stimulation frequency influences the mechanisms of visual selection and attentional capture.

To address this gap, we recruited PD patients with impaired visual attention and investigated the effects of both high-frequency (130 Hz) and theta low-frequency (5 Hz) STN-DBS on their performance in a computerized visual search task. To disentangle the mechanisms underlying attention from those related to motor response execution, we employed a variant of the attentional capture paradigm, complemented by behavioral and electrophysiological measurements. By comparing performance across OFF, high-frequency, and low-frequency stimulation states, we aim to dissociate the frequency-specific influence of STN-DBS on top-down attentional control versus bottom-up stimulus-driven capture.

## Materials and methods

### Study design and participants

We recruited 39 participants with Parkinson’s disease who had undergone bilateral STN-DBS surgery at the department of functional neurosurgery from March 2025 to March 2026. All participants diagnosed with Parkinson’s disease fulfilled the International Parkinson and Movement Disorder Society criteria for Parkinson’s disease and the following inclusion criteria: (i) at least 30% response to acute L-Dopa challenge; (ii) absence of dementia (MMSE > 20 for individuals with 1–6 years of education and MMSE > 24 for individuals with 7 or more years of education); (iii) predominantly akinetic-rigid subtype, with no obvious tremor; (iv) normal or corrected-to-normal vision, and they did not suffer from deficiencies in red-green color vision. Further, we recorded levodopa equivalent daily dose (LEDD), education years, Hoehn and Yahr scale rating (H-Y), disease duration, the Hamilton Anxiety Scale (HAMA), the Hamilton Depression Scale (HAMD), the Mini Mental State Examination (MMSE) and the Montreal Cognitive Assessment (MoCA).

### DBS and stimulation settings

All patients were implanted bilaterally in the STN with stereotactic implantation based on fusion of preoperative 3.0 T MR and frame-based CT scans, microelectrode recording using PINS (quadripolar L301 leads, Beijing) electrodes, according to the surgical procedure described in previous studies (Yin et al., 2021). After the implantable pulse generator (IPG) was placed within the subclavicular pocket under general anesthesia, the bilateral electrodes at the target site were connected to the IPG via extended leads. A postoperative CT scan was instantly performed the day of surgery to ensure the absence of complication and was used for intracranial electrodes reconstruction and localization. Only patients with correct placement of the electrodes in both STNs and in particular with at least one lead contact in the sensorimotor part and another contact in the associative part of the STN entered our protocol.

After DBS implantation, participants performed the same set of tasks under three different bilateral stimulation conditions with a 10 min washout period: no stimulation (OFF), bilateral ventral high-frequency (HF) DBS (130 Hz) and bilateral ventral low-frequency (LF) theta stimulation (5 Hz). The ventral contacts were selected as the stimulation site based on the established functional topography of the STN. Specifically, the ventral portion of the STN is known to receive prominent afferents from, and project back to, the associative and limbic cortices—including the prefrontal cortex and anterior cingulate cortex—whereas the dorsal STN is more closely linked to sensorimotor circuits (Lambert et al., 2012). Given that our study aimed to modulate visual attention—a cognitive function heavily reliant on prefrontal-striatal networks—we chose the ventral contacts to preferentially engage the associative/limbic subregions of the STN. The current was set to 2.0 mA and the pulse width was set to 60 μs based on preclinical data utilizing DBS for memory.

All experimental sessions were conducted 3 days after electrode implantation, during the acute postoperative period. This time window, while introducing potential confounds such as microlesion effects and postoperative fatigue, has been successfully employed in prior intracranial recordings during cognitive tasks (Zhang et al., 2022). The early postoperative setting allows for recordings in a stimulation-naïve state, minimizing the influence of chronic stimulation-induced plasticity. To mitigate potential order effects, the presentation order of the three stimulation conditions was permuted and counterbalanced across participants using a Latin square design. The MDS-Unified Parkinson’s Disease Rating Scale (UPDRS-III) motor examination assessment was performed under each stimulation state. The experiment lasted up to about 1 h, with each experimental stimulation session lasting 10–15 min on average. Participants were instructed to abstain from their regular medication for a minimum of 12 h before testing to eliminate any potential confounding effects on cognitive performance. All experimental sessions were double-blind, with an additional experimenter performing the DBS programming before each session.

### Electrode reconstruction and localization

Electrode reconstruction and localization were performed using the Lead-DBS toolbox (version 3.1) following the pipeline established in previous studies (Fan et al., 2023). Briefly, preoperative T1-weighted MRI and postoperative CT images were linearly co-registered and nonlinearly normalized into the Montreal Neurological Institute (MNI) ICBM 2009b NLIN ASYM template space using the Advanced Normalization Tools (ANTs). Electrode trajectories and contact positions were reconstructed using the PaCER algorithm and manually refined by two experienced neurosurgeons to ensure accuracy. The active contacts were defined as the cathodal contacts used for stimulation. Importantly, the same pair of bilateral ventral contacts was used for both 5-Hz and 130-Hz stimulation conditions, ensuring that frequency-specific effects were not confounded by differences in stimulation location. Electrode contacts were visualized using the DISTAL minimal atlas for anatomical localization. The MNI coordinates of the active contacts were extracted for each patient and each hemisphere.

### Experimental paradigms

The AC tasks were created and run with the Psychtoolbox Version 3.0 in Matlab2018b. During each task, participants were seated ∼50 cm away from a 15-inch monitor. They indicated their responses via two button boxes, connected to the computer via a USB port, marked with an up-arrow symbol for a right-hand response and a down-arrow symbol for a left-hand response. A practice session was administered before each task. The task consisted of three blocks, each comprising 60 trials, yielding a total of 180 trials per session. The two trial types (no-distractor and distractor) were randomly interleaved in equal proportions. For each task, response time and accuracy were recorded.

A schematic of the task is presented in Figure 1. The attentional capture (AC) task was adapted from the paradigm developed by Tommasi et al. (2015). Each trial began with the presentation of a white fixation cross (0.5°) at the center of a black screen, accompanied by an auditory warning signal lasting 300 ms. Subsequently, six green diamond-shaped stimuli (1.2° on a side) appeared, equally spaced around the fixation cross on an imaginary circle with a radius of 3.6°, and remained on the screen for 700 ms. Following this, participants were presented with either a no-distractor trial or a distractor trial. In the no-distractor condition, one of the six green diamonds abruptly had its upper or lower corner cut off (0.6° on a side), thereby becoming a pentagon-shaped target with its base oriented either upward or downward. Participants were required to indicate the orientation of the pentagon’s base: pressing the up-arrow key if the base was at the top or the down-arrow key if it was at the bottom. In the distractor condition, simultaneously with the appearance of the target pentagon, one of the remaining five diamonds abruptly changed its color from green to red and underwent a 45° rotation, becoming a square-shaped singleton distractor. This salient but task-irrelevant stimulus was assumed to capture attention automatically and interfere with target identification. Participants were instructed to maintain fixation on the central cross and to respond exclusively to the target while ignoring the distractor. The stimulus display remained visible for up to 2000 ms, during which participants were required to respond. If no response was made within 2000 ms, or immediately after the participant pressed a response key, the screen turned black, serving as an inter-trial interval of 1,500 ms, after which the next trial began. Reaction time (RT) was defined as the interval from the onset of the target array to the participant’s button press. Only correct-response trials were included in RT analyses, and trials with RTs < 200 ms were excluded as anticipatory responses. Patients were classified into visually impaired attention (VIA) and visually normal attention (VNA) groups based on their accuracy in the distractor condition of the AC task during the OFF-stimulation session, using a 90% accuracy cutoff, consistent with previous studies (Tommasi et al., 2015). This threshold was further validated by ROC analysis.

**Figure 1:**
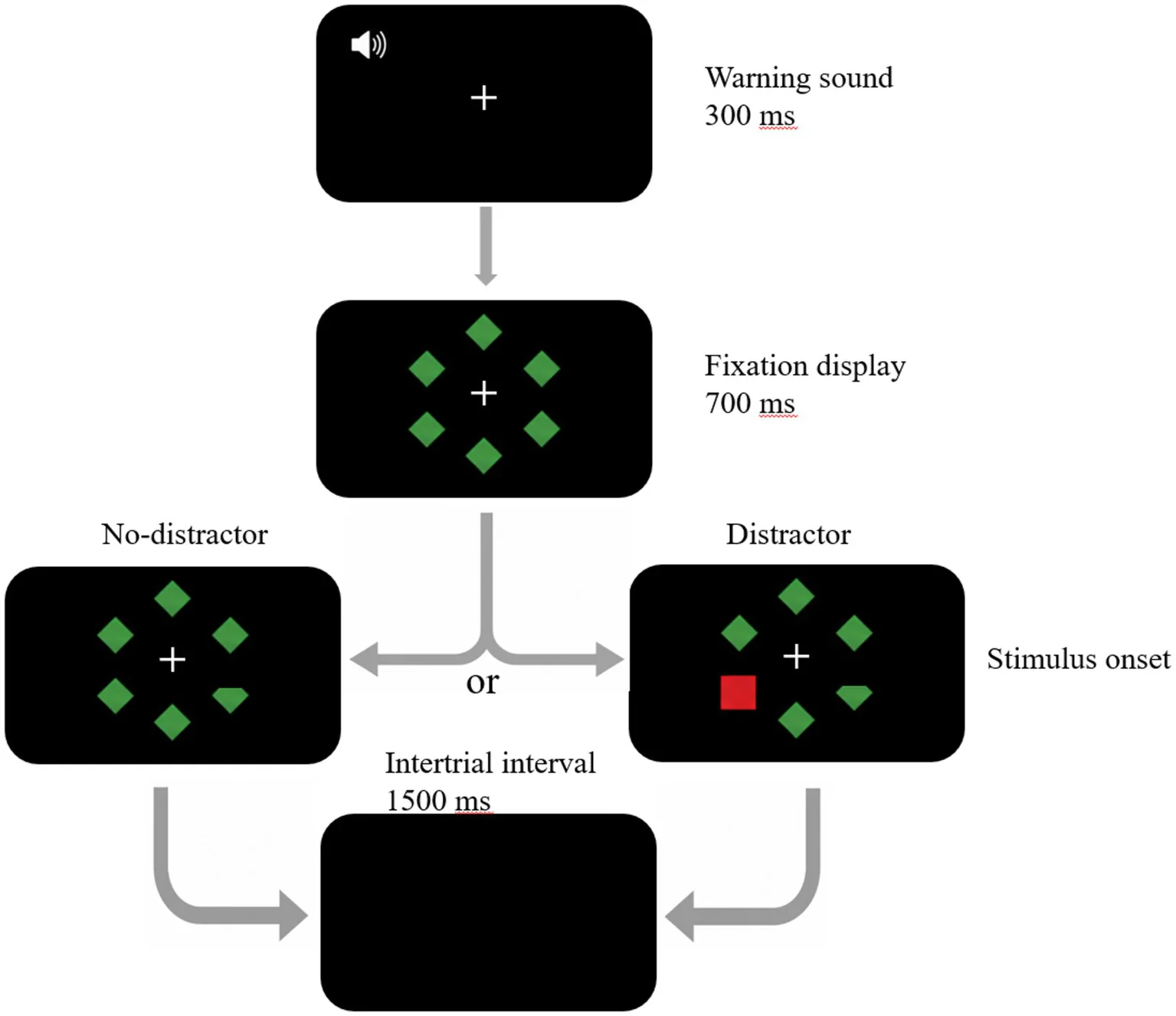
Schematic illustration of the attentional capture (AC) task. Each trial started with a fixation cross (0.5°) and an auditory warning signal (300 ms), followed by six green diamonds (1.2°) equally spaced on an imaginary circle (radius: 3.6°) for 700 ms. In no-distractor trials, one diamond changed into a pentagon with an upward or downward base; participants reported the base orientation via arrow keys. In distractor trials, a task-irrelevant singleton distractor (red square, 45° rotated) appeared simultaneously with the target pentagon among the remaining distractors. The display lasted up to 2000 ms, followed by a 1,500-ms inter-trial interval. The task was adapted from Tommasi et al. (2015).

### EEG recording and preprocessing

Electroencephalographic (EEG) activity was recorded using a 64-channel Ag/AgCl cap according to the extended 10–20 system (10/05 system), with a linked mastoid reference (Nihon Kohden, Tokyo, Japan). The signal was sampled at 1000 Hz and synchronized with the AC task. Electrode impedance was maintained below 10 kΩ. Data were reviewed, clipped, and exported using NeuroWorkbench software (Nihon Kohden, Tokyo, Japan).

Offline preprocessing was performed using EEGLAB 2021.1 running under MATLAB 2022b. Channel labels were corrected, and standard electrode positions were assigned using the 10/05 system. Continuous data were band-pass filtered from 0.1 Hz to 40 Hz, with a 50-Hz notch filter applied to remove line noise. Data were then downsampled to 200 Hz and segmented into epochs ranging from −1,000 ms to 2000 ms relative to stimulus onset, with baseline correction applied from −200 ms to stimulus onset. Bad channels were interpolated, and epochs containing peak-to-peak amplitudes exceeding ±100 μV in any channel were automatically rejected prior to analysis. Across all participants and conditions, the mean number of retained epochs per block was 52.3 ± 6.7 (out of 60) for the OFF condition, 50.1 ± 7.2 for the 5 Hz stimulation condition, and 51.8 ± 6.5 for the 130 Hz stimulation condition. Ocular, muscular, and other physiological artifacts were removed using independent component analysis (ICA).

DBS artifacts were removed using the spike detection and removal modules of the DBSFILT toolbox. Spectral spikes were identified using a frequency-domain Hampel identifier (Allen et al., 2010) (window length = 1 Hz, threshold = 1.5) applied to the averaged FFT spectrum across channels, and the detected frequencies were interpolated using the toolbox’s built-in routine (Lio et al., 2018). In addition, we visually inspected individual ERP waveforms and computed the topographic distribution of the signal-to-noise ratio within the P300 window; no stimulation-frequency-dependent SNR differences were observed, confirming adequate artifact suppression.

### ERP analysis

Epochs were averaged separately for distractor and no-distractor conditions over a time window from −100 ms to 800 ms relative to stimulus onset. The P300 was defined as the most positive peak within the 250–400 ms latency window following stimulus onset. To objectively determine peak latency, we computed the Global Field Power (GFP)—the spatial standard deviation of the potential field across all electrodes—at each time point. The P300 peak was identified as the time point at which GFP reached its maximum within the designated window. Amplitudes were then measured at the midline electrodes (Fz, Cz, and Pz) at each participant’s individual peak latency, defined as the voltage difference between the baseline and the maximal positive peak. Latency was defined as the time elapsed from stimulus onset to the P300 peak.

### Statistical analysis

#### Behavioral data

All statistical analyses were performed using R (version 4.5.1). Data were visualized using the ggplot2 and ggdist packages. Bayesian mixed-effects models were implemented using the brms package to analyze reaction time and accuracy data. For response time analyses, we fitted shifted log-normal regression models to account for the right-skewed distribution of response time. Accuracy data were modeled using beta regression, which requires the dependent variable to be strictly within the open interval (0, 1). Since no accuracy values of zero were observed and only a small proportion (4%) of observations were exactly 1, we applied a minimal boundary adjustment following the standard practice recommended by Smithson and Verkuilen (2006). Specifically, we replaced accuracy values of 1 with 0.999, while leaving all other values unchanged. All models included stimulation condition (OFF, HF, LF) as a fixed effect and participant-specific random intercepts to account for individual variability.

We used hypothesis testing contrasts (hypr package) to compare specific stimulation conditions: HF vs. OFF and LF vs. OFF. Bayes factors (BFs) were calculated using bridge sampling (Gronau et al., 2017) to quantify evidence for or against the inclusion of each effect. BF > 1 indicates evidence in favor of an effect, with evidential strength evaluated according to Jeffreys’ criteria (Kricheldorff et al., 2025): BF > 30 as substantial, 10–30 as strong, 3–10 as moderate, and 1–3 as anecdotal evidence; BF < 1 supports the null hypothesis. Posterior predictive checks were performed to assess model fit.

#### Amplitude and latency data

P300 amplitudes and latencies were analyzed using repeated-measures ANOVAs with stimulation (OFF, LF, HF) and scalp electrode (Fz, Cz, Pz) as within-subject factors. Sphericity violations were corrected using Greenhouse–Geisser, and effect sizes were reported as partial η^2^. Significant main effects of Stimulation were followed by Bonferroni-corrected paired *t*-tests; significant Stimulation × Electrode interactions were explored via simple effect analyses. Statistical significance was set at *p* < 0.05.

## Results

### Baseline characteristics

Baseline characteristics of the 39 participants are given in Table 1.

**Table 1:** Baseline characteristics.

Variable	Mean (SD)
Age (in years)	64.4 (7.6)
Gender	
Male	26
Female	13
Education (in years)	11.9 (4.0)
Disease duration (in years)	9.7 (7.0)
LEDD (in mg)	1026.5 (588.5)
H-Y (med-off)	3.2 (0.8)
H-Y (med-on)	2.4 (0.6)
HAMA	11.4 (7.7)
HAMD	10.5 (7.4)
MMSE	26.2 (2.2)
MoCA	23.0 (3.5)

Clinical characteristics of the patient group are displayed as means, with standard deviations shown in parentheses or counts. LEDD, levodopa-equivalent daily dose; H-Y, Hoehn and Yahr scale rating; HAMA, Hamilton Anxiety Scale; HAMD, Hamilton Depression Scale; MMSE, the Mini Mental State Examination; MoCA, the Montreal Cognitive Assessment.

### Electrode localization and group comparison

Bilateral STN electrodes were successfully reconstructed in all 39 patients (Figures 2A–C). The active contacts were localized to the ventral portion of the STN in both hemispheres. To exclude the possibility that the observed frequency-dependent effects on visual attention were confounded by differences in stimulation location, we compared the MNI coordinates of the active contacts between the VNA (*n* = 18) and VIA (*n* = 21) groups. Independent-samples *t*-tests revealed no significant differences in any coordinate for either hemisphere after Bonferroni correction (Figures 2D–I). For the right hemisphere, the mean coordinates for the VNA and VIA groups were (X: 12.52 ± 0.87 vs. 12.24 ± 0.71, *p*-corrected = 1.000; Y: −14.21 ± 0.82 vs. −14.70 ± 0.82, *p*-corrected = 0.430; Z: −8.95 ± 0.72 vs. −8.83 ± 0.72, *p*-corrected = 1.000), respectively. For the left hemisphere, no significant differences were observed (X: −11.13 ± 1.00 vs. −11.75 ± 0.91, *p*-corrected = 0.304; Y: −14.44 ± 1.02 vs. −14.54 ± 1.11, *p*-corrected = 1.000; Z: −8.86 ± 0.57 vs. −8.85 ± 0.82, *p*-corrected = 1.000). The effect sizes (Cohen’s d) ranged from −0.01 to 0.65, indicating small to moderate differences that did not reach statistical significance. These results confirm that the active contacts were located at comparable positions within the STN between the two groups, ruling out stimulation site differences as a confound for the observed frequency-dependent effects.

**Figure 2:**
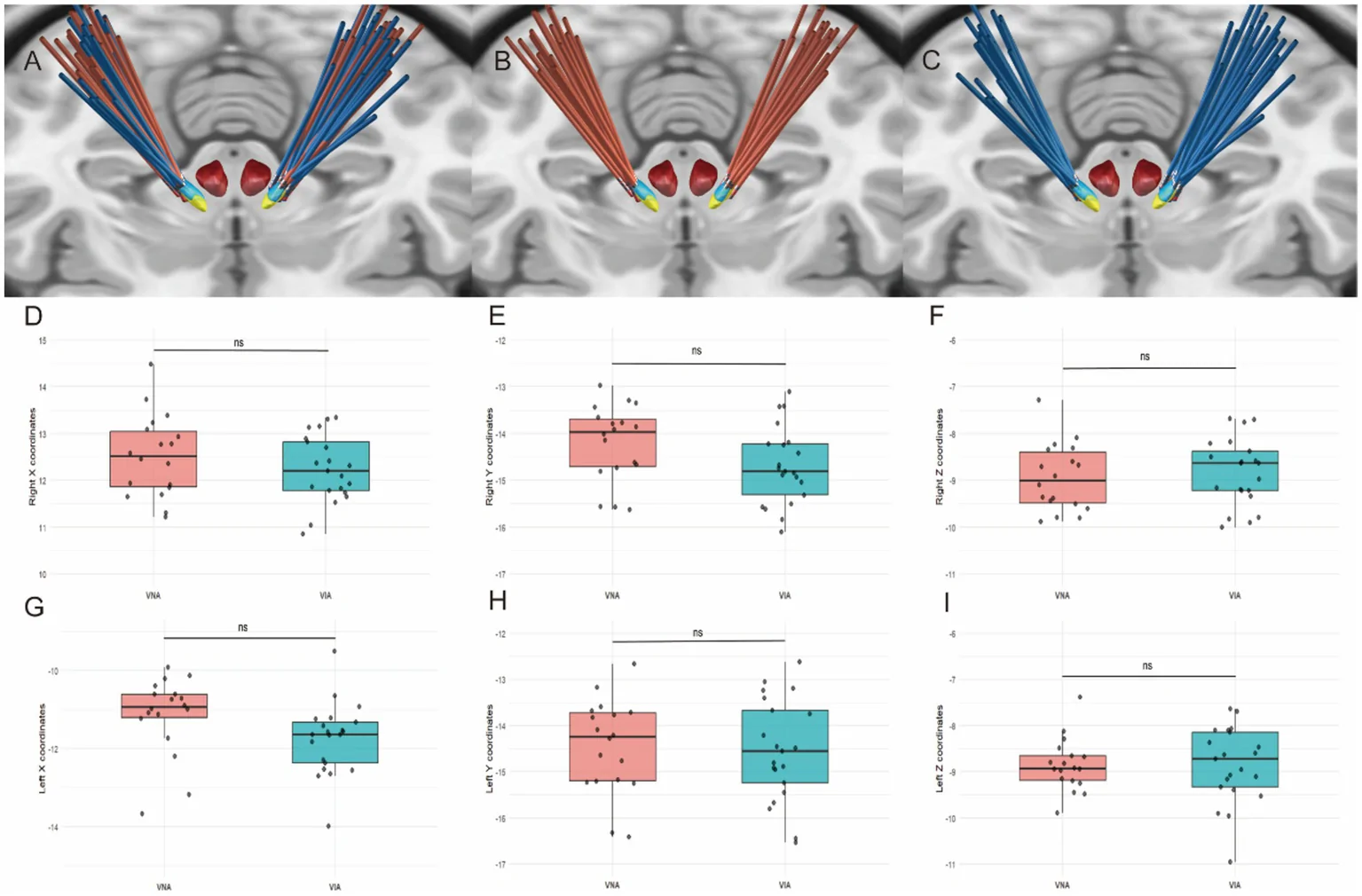
Electrode reconstruction and group comparison of active contact coordinates. **(A)** All 39 patients (both hemispheres) showing the distribution of active contacts (red dots) relative to the STN. light blue, STN associative; yellow, STN limbic; red, red nucleus. **(B)** Subgroup of patients with visually normal attention (VNA, *n* = 18, orange). **(C)** Subgroup of patients with visually impaired attention (VIA, *n* = 21, blue). **(D–I)** Comparison of active contact MNI coordinates between the VNA and VIA groups. Independent-samples *t*-tests revealed no significant differences between the two groups in any coordinate for either hemisphere after Bonferroni correction.

### Behavioral results

#### UPDRS-III

The results revealed a statistically significant main effect of stimulation condition on UPDRS-III scores (*F* (2, 76) = 199.33, *p* < 0.001, partial η^2^ = 0.418), indicating that the scores differed significantly among the three states (Figure 3A). Post-hoc pairwise comparisons with Bonferroni correction further revealed that UPDRS-III scores in the high frequency stimulation were significantly lower than those in the off state (mean difference = 23.36, *t* (38) = 14.29, *p* < 0.001). Similarly, the low frequency condition showed significantly higher scores than the high frequency condition (mean difference = 21.44, *t* (38) = 14.04, *p* < 0.001). Although the difference between off state and low frequency DBS reached statistical significance (mean difference = 1.92, t (38) = 5.33, *p* < 0.001), the magnitude of this improvement was relatively modest compared with the clinically meaningful reduction observed in the high frequency condition (mean difference = 23.36). This suggested that while low frequency stimulation exerted a consistent and statistically significant effect on motor function, its therapeutic benefit may be limited relative to high frequency stimulation.

**Figure 3:**
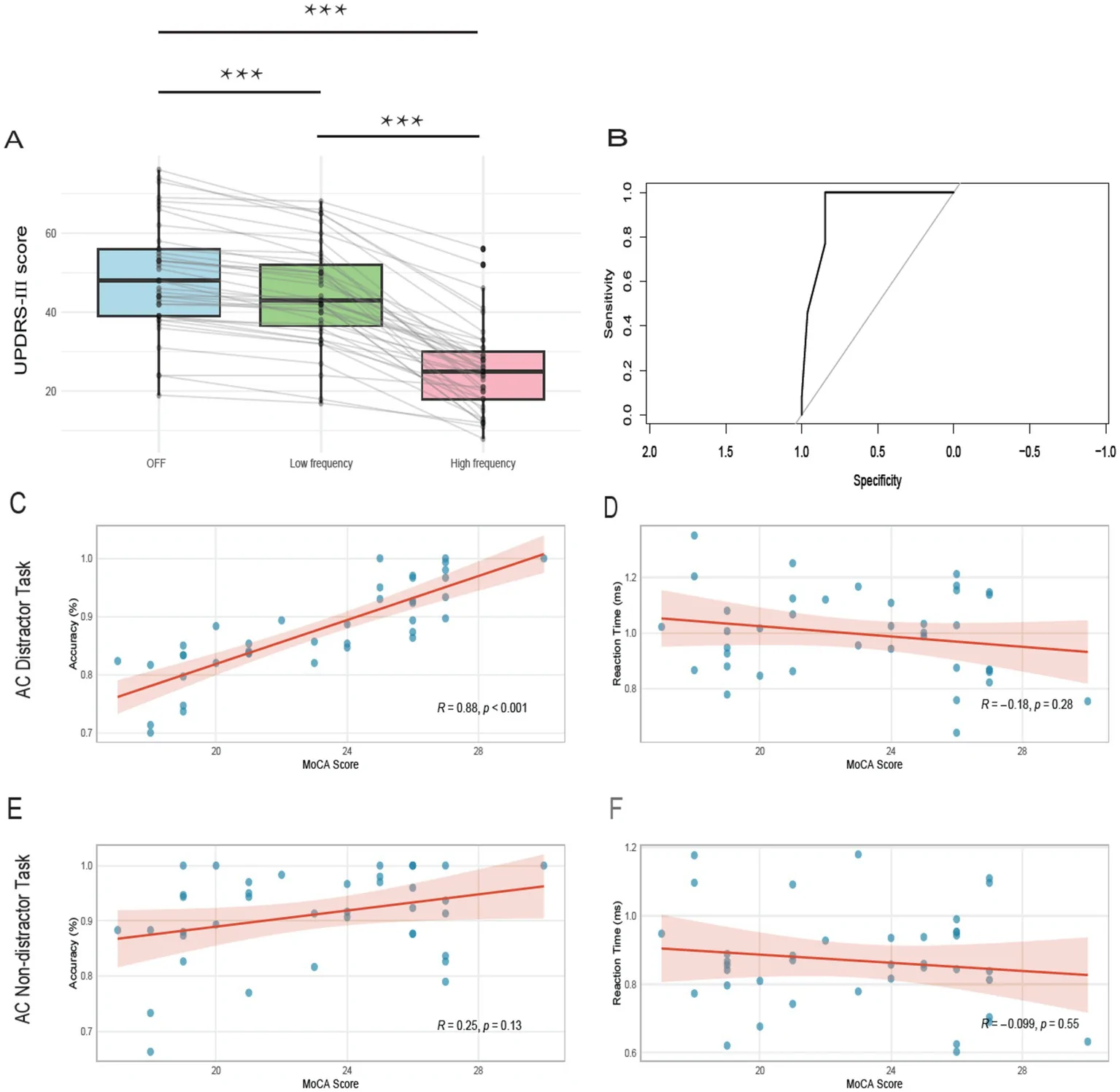
**(A)** UPDRS-III scores across the three stimulation conditions in box plot. Gray dots represent individual participants’ scores; gray lines connect data points from the same participant across conditions. **(B)** ROC curve for MoCA in detecting visual attention impairment. AUC = 0.93 (95% CI: 0.85–1.00). **(C–F)** Correlations between MoCA scores and behavioral performance. Spearman’s correlation coefficients and significance (*p*-value) are shown for accuracy and reaction time in both distractor and non-distractor conditions.

#### AC task results

Spearman’s rank correlation analyses demonstrated a selective association between MoCA scores and visual attention performance. MoCA scores were significantly correlated with task accuracy in distractor paradigm (R = 0.878, *p* < 0.001), whereas no significant correlations were observed with reaction time (R = −0.176, *p* = 0.284). Also, there was no significant correlation between MoCA scores and task accuracy (R = 0.249, *p* = 0.127), or reaction time (R = −0.099, *p* = 0.550) in non-distractor paradigm (Figures 3C–F).

Table 2 presented the demographic and clinical baseline characteristics of the 21 patients with impaired visual attention and the 18 patients with normal visual attention. In addition, to evaluate the clinical utility of the MoCA scale as a screening tool, we conducted a receiver operating characteristic (ROC) curve analysis to determine the MoCA threshold that best distinguished between the two groups. The model showed excellent discriminative ability (AUC = 0.93, 95% CI: 0.85–1.00), with the Youden index identifying a MoCA score of 24.5 as the optimal cutoff, yielding perfect sensitivity (100%) and high specificity (84.6%) (Figure 3B).

**Table 2:** Basic demographic and clinical information about visually impaired attention (VIA) and visually normal attention (VNA) groups.

Variable	VNA (*n* = 18)	VIA (*n* = 21)	*p*
Age (in years)	62.61 ± 8.25	66 ± 6.91	ns
Gender			ns
Male	14 (78%)	12 (57%)	
Female	4 (22%)	9 (43%)	
Education (in years)	12.44 ± 3.7	11.43 ± 4.34	ns
Disease duration (in years)	10.33 ± 9.15	9.24 ± 4.85	ns
LEDD (in mg)	1166.42 ± 791.79	906.52 ± 331.24	ns
H-Y (med-off)	3 ± 0.77	3.38 ± 0.84	ns
H-Y (med-on)	2.33 ± 0.71	2.5 ± 0.47	ns
Clinical improvement	0.48 ± 0.19	0.48 ± 0.14	ns
UPDRS-III (off)	47.56 ± 15.96	50 ± 12.14	ns
UPDRS-III (LF)	46.39 ± 15.48	49.57 ± 12.19	ns
UPDRS-III (HF)	24.72 ± 13.4	26.19 ± 8.78	ns
HAMA	8.22 ± 6.71	14.19 ± 7.83	0.007
HAMD	7.5 ± 6.44	13.1 ± 7.45	0.006
MMSE	27.61 ± 1.69	25.05 ± 1.99	<0.001
MoCA	25.67 ± 2.52	20.76 ± 2.49	<0.001

Data above is provided as mean ± standard deviation or percentage of the full number of patients in the respective group. LEDD, levodopa-equivalent daily dose; H-Y, Hoehn and Yahr scale rating; UPDRS, Unified Parkinson’s disease rating scale; off, stimulation off; LF, low-frequency stimulation; HF, high-frequency stimulation; HAMA, Hamilton Anxiety Scale; HAMD, Hamilton Depression Scale; MMSE, the Mini Mental State Examination; MoCA, the Montreal Cognitive Assessment; ns, non-significant. The clinical improvement is calculated as [UPDRS-III (med-off) – UPDRS-III (med-on)] / UPDRS-III (med-off).

#### Hierarchical Bayesian analysis of behavioral performance

To directly test whether the beneficial effect of 5 Hz stimulation was selective to patients with impaired visual attention and specifically engaged in the distractor-present context, we performed a hierarchical Bayesian analysis. A model including the three-way interaction (Stimulation × Group × Distractor Type) was strongly favored over a model without this interaction (BF = 48.04), indicating that the effect of stimulation frequency on accuracy was jointly dependent on both attention group and distractor presence.

To decompose this three-way interaction, we conducted separate Bayesian model comparisons for the distractor-present and no-distractor conditions. In the distractor-present condition, the Stimulation × Group interaction was overwhelmingly supported (BF = 1.15 × 10^7^), indicating that the effect of stimulation frequency on distractor suppression differed dramatically between VIA and VNA groups. In contrast, the same interaction received substantially weaker support in the no-distractor condition (BF = 17.50). This marked discrepancy confirms that the selective benefit of 5 Hz stimulation was specifically tied to the suppression of salient distractors.

To further characterize the pattern driving these interactions, we examined the simple effect of 5 Hz stimulation (LF vs. OFF) within each combination of group and distractor condition. In the VIA group, 5 Hz stimulation produced a substantial accuracy improvement in the distractor-present condition (BF = 1.84 × 10^11^), whereas no such improvement was observed in the no-distractor condition (BF = 1.26). In the VNA group, 5 Hz stimulation showed no effect in the distractor-present condition (BF = 2.88), but unexpectedly reduced accuracy in the no-distractor condition (BF = 6.31). These results confirm that the selective benefit of theta-frequency stimulation is specific to patients with impaired visual attention and specifically engaged when top-down control is challenged by salient distractors.

Consistent with the above interaction analyses, within the VIA group, 5 Hz stimulation significantly improved accuracy in the AC-distractor task compared with OFF (m = 10.8, 95% CI = [8.4, 13.4%], BF > 1,000), whereas high-frequency stimulation impaired performance (m = −5.5, 95% CI = [−8.5, −2.5%], BF = 122.8). In the VNA group, the AC-distractor task revealed moderate evidence for reduced accuracy under high-frequency DBS (m = −2.7, 95% CI = [−5.3, −0.5%], BF = 6.3), but no evidence for an accuracy difference under low-frequency stimulation (m = −2.1, 95% CI = [−4.6, −0.01%], BF = 2.2).

In the AC-nondistractor task, the VIA group showed moderate evidence for reduced accuracy under high-frequency DBS (m = −3.6, 95% CI = [−6.7, −0.7%], BF = 6.6), but no effect of low-frequency stimulation (m = 2.2, 95% CI = [−0.001, 4.7%], BF = 1.8). In the VNA group, no evidence was found for an accuracy decline under high-frequency DBS (m = −0.7, 95% CI = [−2.2, 0.8%], BF = 0.5); however, moderate evidence supported an accuracy reduction under low-frequency stimulation (m = −1.9, 95% CI = [−3.8, −0.4%], BF = 6.2).

In contrast to the accuracy results, Bayesian analyses of reaction times revealed no reliable evidence of DBS-induced changes across all groups and conditions. In the VIA group, we observed only anecdotal evidence for a slowing under high-frequency stimulation in the distractor condition (m = 51.1 ms, 95% CI = [13.4 ms, 88.3 ms], BF = 2.6), with no evidence for an effect under low-frequency stimulation in either the distractor (m = −6.3 ms, 95% CI = [−42.7 ms, 29.9 ms], BF = 0.1) or no-distractor (m = −31.0 ms, 95% CI = [−65.2 ms, 3.1 ms], BF = 0.3) condition. Similarly, in the VNA group, reaction time analyses yielded no evidence of DBS-related changes under any condition (all BF < 0.6). Given the absence of systematic effects, no hierarchical interaction analyses were performed for reaction time data. These findings indicate that the frequency-dependent effects of STN-DBS were specific to response accuracy, without commensurate changes in processing speed. The behavioral performances are shown in Figure 4.

**Figure 4:**
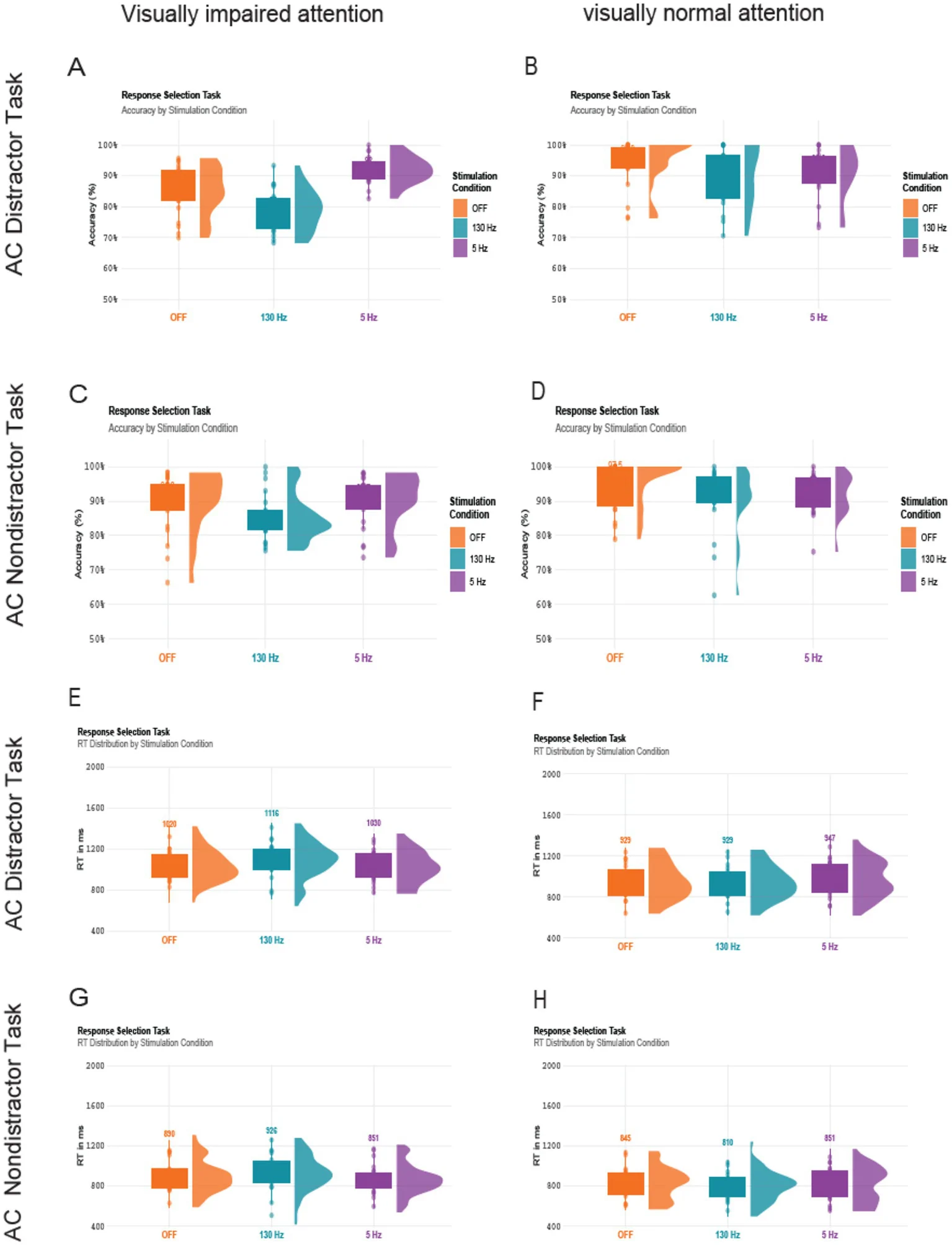
Behavioral performance under different DBS frequencies in VIA and VNA groups. The plots show the distribution of accuracy **(A–D)** and reaction time **(E–H)** across OFF (orange), 130 Hz (teal), and 5 Hz (purple) stimulation conditions. In the AC-distractor task, 5 Hz stimulation significantly restored accuracy in patients with visual attention impairment, while 130 Hz stimulation negatively impacted accuracy in both groups (especially the impaired group). In the AC-nondistractor task, DBS effects were more modest. Reaction time distributions showed no reliable evidence of stimulation-induced changes across groups or conditions.

#### Sensitivity analysis with MoCA as a covariate

To assess whether the observed frequency-dependent effects were confounded by baseline differences in global cognitive status, we repeated our hierarchical Bayesian analyses with MoCA total score as a continuous covariate, while retaining the full three-way interaction structure (Stimulation × Group × Distractor Type) and all lower-order terms. The critical three-way interaction remained strongly supported after controlling for MoCA (BF = 36.71; original: 48.04). Decomposition of this interaction within the covariate-adjusted model revealed that, in the distractor-present condition, the Stimulation × Group interaction continued to receive overwhelming support (BF = 8.26 × 10^6^; original: 1.15 × 10^7^), whereas in the no-distractor condition it received only weak support (BF = 12.89; original: 17.50). Simple effect analyses confirmed that, in the VIA group, the 5 Hz benefit (LF vs. OFF) in the distractor-present condition remained decisive (BF = 1.52 × 10^10^; original: 1.84 × 10^11^), with a nearly identical improvement (m = 10.5, 95% CI = [8.1, 13.2%]) compared with the unadjusted model (m = 10.8, 95% CI = [8.4, 13.4%]). In the VNA group and the no-distractor condition, no meaningful effects emerged after covariate adjustment (all BFs < 3), consistent with the original findings.

Within-group comparisons with MoCA adjustment further corroborated the original results: in the VIA group, LF stimulation significantly improved accuracy in the distractor-present condition compared with OFF (BF > 1,000; original: > 1,000), whereas HF stimulation impaired performance (BF = 98.2; original: 122.8). In the VNA group, no evidence for a LF effect was found in the distractor-present condition (BF = 2.0; original: 2.2), while HF stimulation showed moderate evidence for reduced accuracy (BF = 5.8; original: 6.3). All other comparisons remained non-significant (all BFs < 3). Additionally, a Bayesian correlation between the individual 5-Hz benefit magnitude [Δ Accuracy = Accuracy (Low-frequency) – Accuracy (OFF)] and MoCA scores in the VIA group yielded BF₀₁ = 14.02, providing strong evidence against a relationship between stimulation benefit and baseline global cognition. Collectively, these sensitivity analyses demonstrate that the selective 5 Hz attentional enhancement in VIA patients is robust to the inclusion of MoCA as a covariate, ruling out the alternative explanation that the effect merely reflects differences in global cognitive capacity.

#### Correlation between UPDRS-III improvement and behavioral changes

To assess whether the observed behavioral effects were confounded by stimulation-induced motor improvement, we performed Spearman’s rank correlation analyses between UPDRS-III changes and changes in accuracy and reaction time, separately for each stimulation frequency, task condition, and group. Across all comparisons, no significant correlations were observed in either the distractor or no-distractor condition, in either the VIA or VNA group (all *p* > 0.10). These findings suggest that the frequency-dependent effects of STN-DBS on visual attention performance were independent of concomitant motor improvement.

### ERP analysis

#### P300 amplitude

Of the 39 participants enrolled in the behavioral study, scalp electroencephalography data were successfully recorded from 26 individuals. Table 3 shows the mean P300 amplitudes and latencies in Fz, Cz and Pz for AC distractor task. Figure 5 shows grand averages of ERP waveforms for each stimulus type in Fz, Cz and Pz, with identification of the distracter-elicited P300 components in the VIA group.

**Table 3:** AC distracter task-elicited P300 amplitudes and latencies: mean (standard deviation).

Measure	Group	Condition	AC-distractor task		
			Fz	Cz	Pz
Amplitude (μV)	VIA	OFF	2.76 (1.12)	2.58 (1.31)	2.45 (1.02)
		LF	5.72 (2.50)	5.68 (2.21)	4.40 (1.92)
		HF	0.85 (0.48)	0.77 (0.32)	0.81 (0.39)
	VNA	OFF	1.42 (2.08)	1.71 (2.19)	1.36 (2.02)
		LF	0.74 (1.87)	0.37 (1.51)	0.02 (0.26)
		HF	1.35 (1.43)	1.39 (1.41)	0.58 (0.98)
Latency (ms)	VIA	OFF	315 (28)	316 (30)	320 (35)
		LF	350 (24)	348 (26)	350 (30)
		HF	298 (27)	301 (28)	306 (33)
	VNA	OFF	331 (35)	334 (35)	325 (37)
		LF	322 (35)	319 (36)	311 (33)
		HF	324 (29)	328 (30)	330 (34)

VIA, visually impaired attention; VNA, visually normal attention; OFF, no stimulation; LF, low frequency stimulation; HF, high frequency stimulation.

**Figure 5:**
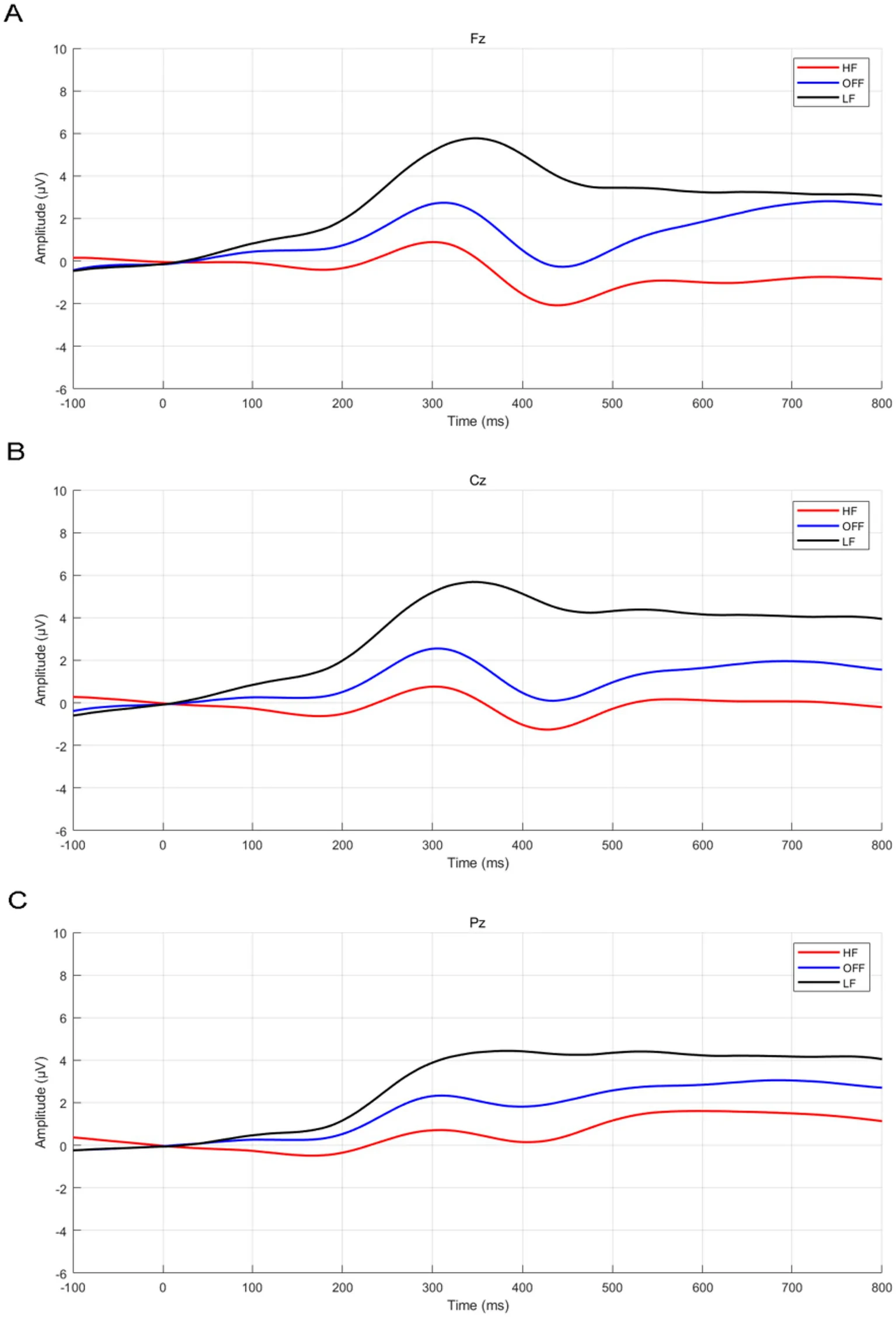
Distracter-elicited grand-averaged P300 event-related potentials (ERPs) at Fz **(A)**, Cz **(B)**, and Pz **(C)** under different DBS conditions in VIA group. Traces represent OFF (blue), low-frequency (black), and high-frequency (red) stimulation in the VIA group. Low-frequency stimulation significantly enhanced P300 amplitudes across all sites compared to OFF (all *p* < 0.05), with a centro-frontal dominant scalp distribution. Conversely, high-frequency stimulation produced a significant, generalized suppression of P300 amplitude compared to both OFF and low-frequency (all *p* < 0.01). Moreover, low-frequency significantly prolonged, whereas High-frequency significantly shortened, P300 latency independent of electrode position (*p* < 0.001).

The repeated-measures ANOVA revealed a significant main effect of “stimulus type” (*F* (2, 26) = 21.89, *p <* 0.001, η^2^ = 0.485) and scalp “electrode position” (*F* (2, 26) = 3.71, *p =* 0.038, η^2^ = 0.016), as well as a significant stimulation × electrode interaction (*F* (4, 52) = 3.77, *p* = 0.009, η^2^ = 0.020) in the VIA group, implying that different frequencies of DBS stimulation significantly modulated the P300 amplitudes, and the modulatory effect of stimulation frequency on amplitudes varied across scalp electrode sites.

Bonferroni-corrected comparisons revealed that at all three electrodes (Fz, Cz, and Pz), the P300 amplitude during low-frequency stimulation was significantly greater than that during the OFF condition (all *p* < 0.05). In contrast, high-frequency stimulation elicited a significantly smaller P300 amplitude compared to both OFF and LF conditions (all *p* < 0.01), indicating a marked amplitude suppression effect.

Bonferroni-corrected pairwise comparisons of P300 amplitudes across scalp electrodes were performed under each stimulation condition. Under the off-state, no significant differences in amplitude were observed among the Fz, Cz, and Pz electrodes (all *p* > 0.05), indicating a uniform scalp distribution. Under low-frequency stimulation, amplitudes at the Fz and Cz electrodes were significantly larger than that at Pz (t (13) = 2.53 and 2.90, respectively; both *p* < 0.05), with no significant difference between Fz and Cz. This suggested that low-frequency stimulation induced a centro-frontal dominant distribution pattern of the P300. Under high-frequency stimulation, no significant differences were found among the three electrodes (all *p* > 0.05), indicating that high-frequency stimulation produced a generalized suppression of the P300 amplitude, eliminating electrode-specific effects.

#### P300 latency

For P300 latency, a repeated-measures ANOVA with stimulation factors and electrode sites revealed a significant main effect of stimulation type (*F* (2, 26) = 11.28, *p <* 0.001, η^2^ = 0.316) in the VIA group. Neither the main effect of scalp electrode sites (*F* (2, 26) = 2.32, *p* = 0.118) nor the stimulation × electrode sites interaction (*F* (4, 52) = 0.86, *p* = 0.493) reached statistical significance. Post-hoc pairwise comparisons with Bonferroni correction for the main effect of stimulation type showed that low-frequency stimulation significantly prolonged P300 latency compared to both off state (t (41) = −5.03, *p* < 0.001) and high-frequency (t (41) = 8.39, *p* < 0.001). Additionally, high-frequency stimulation significantly shortened latency relative to OFF (t (41) = 2.66, *p* = 0.033). These results indicated that stimulation frequency exerted a significant modulatory effect on P300 latency, with low-frequency producing a marked prolongation and high-frequency producing an acceleration of the latency, independent of electrode site.

Repeated-measures ANOVA was also performed to examine the effects of stimulus type and electrode positions on P300 latency and amplitude in the VNA group. For latency, no significant main effects or interaction were observed (stimulation: *F* (2, 22) = 0.69, *p* = 0.513; electrode: *F* (2, 22) = 1.59, *p* = 0.227; stimulation × electrode: *F* (4, 44) = 1.36, *p* = 0.262). For amplitude, similarly, no significant effects were found (stimulation: *F* (2, 22) = 1.35, *p* = 0.281; electrode: *F* (2, 22) = 3.24, *p* = 0.059; stimulation × electrode: *F* (4, 44) = 0.73, *p* = 0.578). Collectively, these results indicated that DBS frequency did not exert significant modulatory effects on P300 latency or amplitude in the normal cohort.

For the AC non-distractor paradigm, visual inspection of the grand-averaged and individual ERP waveforms revealed no identifiable P300 component at any electrode site (Fz, Cz, Pz) in any participant. Specifically, no reliable positive deflection was observed within the typical 250–400 ms latency window. Therefore, statistical analyses—including repeated-measures ANOVAs and post-hoc comparisons—were not performed on the P300 data from this condition. This absence of a robust P300 response suggests that the non- distractor task did not sufficiently engage the cognitive processes necessary to generate this endogenous component.

## Discussion

In this study, we investigated the effect of bilateral 130 Hz and 5 Hz STN-DBS stimulation on visual attention in PD patients. To this end, we performed AC-distractor or nondistractor task under high-frequency, low-frequency and no stimulation after DBS. Our results indicated that PD patients with impaired visual attention function showed increased accuracy in AC-distractor task under theta low-frequency DBS compared with off state, suggesting low-frequency stimulation can suppress distractor. The effect of low-frequency DBS was limited to control inhibition, as we found no effect on simple visual attention in AC-nondistractor task. Notably, in PD patients with intact visual attention, neither DBS frequency significantly altered the ability to suppress distractor interference compared with the off-state, contrasting with the pronounced effects observed in the impairment group. In line with our results, Kricheldorff *et al.* have reported similar behavioral findings when comparing low and high-frequency DBS using other paradigms (Kricheldorff et al., 2025).

### AC-task

The attentional capture (AC) task served as the primary behavioral tool in the present study. In this task, participants were instructed to search for a pentagon-shaped target embedded among five diamonds and to report the orientation of its base (top or bottom) via keypress. On distractor-present trials, one of the non-target diamonds abruptly changed both its color and orientation, thereby acting as a highly salient singleton distractor presumed to capture attention automatically via bottom-up mechanisms. Importantly, performance in the AC task reflected not only the interplay between top-down and bottom-up attentional control mechanisms, but also other component processes, including response selection and motor response initiation. This compound search design allowed us to disentangle factors affecting target selection from those influencing subsequent response selection, as the response requirements remained constant across distractor and no-distractor conditions.

A critical methodological consideration concerns the use of covert versus overt attention. Our paradigm was designed to engage covert attention by instructing participants to maintain central fixation throughout each trial, such that attentional deployments to discrete locations would occur without overt eye movements. This design choice was particularly important given that PD patients are known to exhibit fundamental motor deficits, including impaired saccadic control (Terao et al., 2011). Had we employed an oculomotor version of the capture task, it would remain uncertain whether the findings truly indexed attentional selection or instead reflected overt motor behavior. However, we acknowledge that without concurrent eye-tracking, we cannot completely rule out the contribution of microsaccades or small gaze shifts. Therefore, our interpretation assumes that participants largely complied with the fixation instructions, and the observed effects should be viewed as primarily—though not exclusively—reflecting covert attentional mechanisms.

### Pathophysiological mechanisms of visual attention deficits in PD

Previous studies have established the basal ganglia as a critical node in the neural architecture subserving visual attention. In Parkinson’s disease, disruption of the cortico-basal ganglia loops is thought to disrupt the delicate equilibrium between bottom-up (exogenous) and top-down (endogenous) processing streams, ultimately giving rise to characteristic visual attention deficits (Esposito et al., 2021).

In the present study, PD patients with impaired visual attention exhibited significantly higher error rates in the AC-distractor condition, unaccompanied by any commensurate increase in reaction times—a pattern that strongly implicated a speed-accuracy trade-off, wherein participants might prioritize response speed over accuracy depending on task demands, instructions, and contextual factors. Critically, this observation provided unambiguous evidence that the pathological state modulated the mechanisms subserving attentional capture, while definitively excluding any confounding contribution of DBS lead implantation to these effects.

Beyond the speed-accuracy trade-off account, the absence of RT shortening—particularly under 130 Hz stimulation, which produced a 23-point improvement in UPDRS-III scores—merits further consideration. Several factors may account for this apparent dissociation. First, RT reflects not only motor execution but also perceptual, attentional, and decision-making processes; the P300 suppression observed under high-frequency stimulation suggests that this frequency may disrupt higher-order cognitive processing, potentially offsetting the motor-speed gain. Second, the UPDRS-III assesses a broad range of motor functions (e.g., limb bradykinesia, rigidity, gait), whereas the present task requires only a simple finger press; the relationship between global motor improvement and fine manual RT is therefore not necessarily linear. Consistent with this interpretation, our correlational analyses revealed no significant association between UPDRS-III changes and RT changes under any stimulation condition (all *p* > 0.10), further supporting the view that the observed behavioral effects were largely independent of concomitant motor improvement.

The enhanced distractibility observed in our patients when confronted with irrelevant yet salient stimuli is fully consistent with prior reports in the PD literature (Uc et al., 2007; Righi et al., 2023; Wang et al., 2025; Norton et al., 2016). In a controlled study of medication-withdrawn PD patients performing a visuospatial memory task, both behavioral and electroencephalographic measures revealed severe deficits in distractor filtering (Lee et al., 2010). Likewise, other studies demonstrated that a substantial proportion of non-demented PD patients, relative to healthy controls, exhibited impaired perceptual discrimination of simple visual stimuli when presented alongside competing, task-irrelevant information (Pilgrim et al., 2021; Redinbaugh and Saalmann, 2024). Collectively, these converging lines of evidence support the view that the observed effects are likely attributable to a relative weakening of top-down attentional control, thereby advancing our understanding of the functional role of cortico-basal ganglia circuits in visual attention.

### Low-frequency and high-frequency stimulation effects

The present study investigated the differential effects of low-frequency (5 Hz) and high-frequency (130 Hz) DBS of the subthalamic nucleus on visual attention in PD patients. Our behavioral findings revealed a dissociation between stimulation frequencies across attention tasks, while ERP measures provided neural correlates that illuminate the underlying mechanisms.

In the VIA group, theta-frequency DBS significantly improved accuracy in the AC-distractor task compared to off-state, whereas high-frequency DBS impaired performance. This pattern was accompanied by increased P300 amplitude under theta stimulation and decreased P300 amplitude under high-frequency stimulation, suggesting that theta-frequency DBS enhanced attentional resource allocation to task-relevant stimuli, while high-frequency stimulation disrupted this process. The prolongation of P300 latency under theta stimulation further indicated extended cognitive evaluation time, consistent with the notion that theta-frequency DBS might promote more deliberate stimulus processing, thereby improving accuracy at the expense of processing speed.

These findings align with a growing body of evidence demonstrating frequency-dependent effects of STN-DBS on cognitive functions. Kricheldorff et al. (2025) reported that 20 Hz beta stimulation improved automatic response inhibition in PD patients, as evidenced by increased accuracy and slowed responding on uncertain Go trials. Similarly, our theta-frequency stimulation enhanced accuracy in the distractor condition, a task requiring suppression of salient distractors and engagement of top-down attentional control. The P300 amplitude enhancement we observed under theta stimulation corroborated the notion that low-frequency stimulation might facilitate cognitive processing by modulating decision thresholds (Wiecki and Frank, 2013), allowing participants to accumulate more evidence before responding, thereby improving accuracy.

Conversely, the detrimental effects of high-frequency stimulation on attentional performance reflected its known propensity to impair cognitive flexibility and response inhibition (Jahanshahi, 2013). The generalized suppression of P300 amplitude across all electrode sites under high-frequency stimulation in our study provided direct neural evidence for this cognitive impairment, suggesting that high-frequency DBS would disrupt the neural networks subserving attentional processing. This is consistent with the observation that high-frequency stimulation, while effective for motor symptoms, can compromise cognitive functions by interfering with cortico-basal ganglia loops involved in attention and cognitive control (Aron et al., 2016).

Notably, in the VNA group, neither behavioral nor ERP measures showed significant DBS-induced modulation. This finding suggested that the frequency-dependent effects of STN-DBS on attention depended on the integrity of attentional networks. Patients with preserved visual attention possessed sufficient compensatory mechanisms to maintain performance regardless of stimulation frequency, whereas those with pre-existing attentional deficits were more vulnerable to stimulation-induced modulation. This interpretation was supported by the absence of identifiable P300 components in the non-distractor condition across all groups, indicating that the cognitive demands of the task were insufficient to engage the neural generators of this endogenous component. The scalp topographical changes we observed—specifically the centro-frontal distribution of P300 under theta stimulation—reflected the engagement of prefrontal-striatal circuits critical for top-down attentional control (Ferrante et al., 2018). The inferior frontal gyrus and pre-supplementary motor area, both connected to the STN via the hyperdirect pathway (Aron et al., 2014), were known to be involved in attentional control and response inhibition. The enhanced P300 amplitude over frontal and central regions under theta stimulation therefore indexed improved top-down modulation of visual attention, consistent with the behavioral improvement observed in the distractor task.

These findings have important clinical implications. While high-frequency DBS remains the standard for motor symptom management, the cognitive costs associated with this treatment are increasingly recognized. Our results suggest that theta-frequency stimulation, either alone or as an adjunct to high-frequency stimulation, offers a means to preserve or even improve attentional function in PD patients with visual attention deficits. However, the beneficial effects are confined to the impaired group, underscoring the importance of patient stratification based on cognitive status when considering novel stimulation protocols.

At the same time, it is important to note that the impaired visual-attention group also exhibited lower MoCA scores and higher anxiety and depression scores than the normal group, raising the possibility that the observed DBS effects might reflect a general cognitive or neuropsychiatric vulnerability rather than a specific attentional improvement. However, several lines of evidence argue against this non-specific interpretation. First, visual attention is a core domain of cognition, and its impairment inherently correlates with global cognitive decline; the group differences in MoCA thus likely reflect the same underlying attentional deficit captured by our task. Second, and more critically, the beneficial effect of 5 Hz stimulation was strictly task- and condition-specific: it emerged exclusively in the distractor-present condition of the AC task, with no significant improvement in the distractor-absent condition, and no commensurate changes in reaction time. This pattern of selective modulation—enhancing accuracy only when top-down control is challenged by salient distractors—strongly supports a specific effect on attentional capture and distractor suppression, rather than a non-specific enhancement of general cognitive or affective state.

Nevertheless, several limitations warrant consideration. First, the relatively small sample size limits the generalizability of our findings. Second, testing was conducted at day 3 post-implantation, an acute period in which microlesion effects, edema, and fatigue may confound performance independently of stimulation. Although we counterbalanced conditions and this time window has precedent (Zhang et al., 2022), our findings should be viewed as acute effects requiring longitudinal validation. Third, the absence of local field potential recordings precludes direct examination of how stimulation frequencies modulate STN oscillatory activity. Future studies combining behavioral measures, ERP, and intracranial recordings will be essential to elucidate the neural mechanisms underlying frequency-dependent effects of STN-DBS on visual attention. Additionally, investigation of adaptive DBS strategies that combine frequencies optimized for both motor and cognitive outcomes represents a promising avenue for future research (Jia et al., 2024).

## Conclusion

The present study demonstrates that theta-frequency (5 Hz) STN-DBS selectively improves visual attention in PD patients with pre-existing attentional deficits, whereas high-frequency (130 Hz) stimulation impairs performance. These behavioral effects are paralleled by frequency-specific modulation of P300 amplitude and latency, suggesting that low-frequency stimulation enhances top-down attentional resource allocation, while high-frequency stimulation disrupts the neural networks subserving attentional control. Our findings provide novel insights into the frequency-specific role of the STN in visual attention and highlight the potential of theta-frequency stimulation as a complementary approach to preserve cognitive function in PD patients undergoing DBS therapy. Future studies combining behavioral, electrophysiological, and intracranial recordings are warranted to further elucidate the underlying neural mechanisms and to explore adaptive DBS strategies for optimizing both motor and cognitive outcomes.

## Data Availability

The raw data supporting the conclusions of this article will be made available by the authors, without undue reservation.
